# Reproducibility of Gadolinium Enhancement Patterns and Wall Thickness in Hypertrophic Cardiomyopathy

**DOI:** 10.5935/abc.20160087

**Published:** 2016-07

**Authors:** Gaston A. Rodriguez-Granillo, Alejandro Deviggiano, Carlos Capunay, Macarena C. De Zan, Patricia Carrascosa

**Affiliations:** Department of Cardiovascular Imaging - Diagnóstico Maipú, Buenos Aires - Argentina

**Keywords:** Cardiomyopathy, Hypertrophic, Reproducibility of Results, Gadolinium, Ventricular Dysfunction, Magnetic Resonance Imaging

## Abstract

**Background:**

Reproducibility data of the extent and patterns of late gadolinium enhancement (LGE) in hypertrophic cardiomyopathy (HCM) is limited.

**Objective:**

To explore the reproducibility of regional wall thickness (WT), LGE extent, and LGE patterns in patients with HCM assessed with cardiac magnetic resonance (CMR).

**Methods:**

The extent of LGE was assessed by the number of segments with LGE, and by the total LV mass with LGE (% LGE); and the pattern of LGE-CMR was defined for each segment.

**Results:**

A total of 42 patients (672 segments) with HCM constituted the study population. The mean WT measurements showed a mean difference between observers of -0.62 ± 1.0 mm (6.1%), with limits of agreement of 1.36 mm; -2.60 mm and intraclass correlation coefficient (ICC) of 0.95 (95% CI 0.93-0.96). Maximum WT measurements showed a mean difference between observers of -0.19 ± 0.8 mm (0.9%), with limits of agreement of 1.32 mm; -1.70 mm, and an ICC of 0.95 (95% CI 0.91-0.98). The % LGE showed a mean difference between observers of -1.17 ± 1.2 % (21%), with limits of agreement of 1.16%; -3.49%, and an ICC of 0.94 (95% CI 0.88-0.97). The mean difference between observers regarding the number of segments with LGE was -0.40 ± 0.45 segments (11%), with limits of agreement of 0.50 segments; -1.31 segments, and an ICC of 0.97 (95% CI 0.94-0.99).

**Conclusions:**

The number of segments with LGE might be more reproducible than the percent of the LV mass with LGE.

## Introduction

The extent of late gadolinium enhancement (LGE) in cardiac magnetic resonance (CMR) as an expression of underlying myocardial fibrosis has been consistently established as an independent predictor of ventricular dysfunction, complex arrhythmias, and death in diverse population settings, particularly in patients with hypertrophic cardiomyopathy (HCM).^1-4^

Over the past decade, implantable cardioverter-defibrillators (ICDs) have demonstrated to be effective in the primary prevention of sudden cardiac death (SCD) in patients with HCM. Notwithstanding, current risk stratification algorithms fail to identify a significant number of patients at risk of SCD deemed at low risk, possibly due to a large heterogeneity in the phenotypic expression and the myriad of genes involved in this disease.^5^

In a recent large cohort of patients with HCM who underwent LGE-CMR, the extent of fibrosis was independently associated to an increase in SCD and to the development of end-stage HCM.^3^ Maximum wall thickness (WT) and percent of left ventricle (LV) with LGE (% LGE) are, respectively, established and emerging risk factors for SCD. Indeed, the % LGE has emerged as a variable with a continuous relationship with the risk of SCD. Nevertheless, reproducibility data of % LGE in HCM is limited, and there is a lack of data in this population regarding reproducibility patterns of LGE and regional WT. Given the wide range of prevalence of LGE in HCM reported in the literature (ranging from 40 to 80%), these data are pivotal for the internal validation aimed at improving risk stratification strategies, and also to establish threshold levels above which longitudinal changes might be significant.^2,3,6,7^

## Methods

### Study population

The objective of this observational study is to explore the reproducibility of regional wall thickness (WT), % LGE, and LGE patterns in patients with HCM. To that end, we retrospectively searched our CMR database from September 2013 to September 2014 and selected patients with confirmed or suspected HCM referred to our institution for LGE-CMR evaluation. Patients with moderate to severe valvular heart disease were excluded, as well as patients with known ischemic cardiomyopathy and those who had underwent septal myectomy or percutaneous septal alcohol ablation.

### CMR acquisition

All CMR exams were performed using the same system (Achieva 1.5 Tesla, Philips Healthcare, Cleveland, OH). A five-element cardiac phased-array coil was used for signal reception and cardiac synchronization was performed using a vector electrocardiogram. Cine-CMR images were acquired in 8-10 contiguous short-axis slices from the level of the mitral valve annulus through the LV apex using a commercially available steady-state free precession pulse sequence. Technical parameters were as follows: TR/TE (ms): 3.5/1.8; flip angle: 60°; section thickness: 8 mm; matrix: 144 x 157; field of view: 320 mm; voxel size: 2.2 x 2.0 mm; and number of phases: 30. For detection of the presence, extent and location of fibrosis, a breath-hold, T1-weighted, contrast-enhanced inversion-recovery segmented gradient echo sequence (TR/TE (ms): 4.8/2.3; flip angle: 25°; section thickness: 10 mm; matrix: 184 x 154; field of view: 320 mm; voxel size: 1.75 x 1.95 mm) was used. These LGE-CMR images were acquired 10 minutes after intravenous administration of 0.2 mmol/kg of a commercially available gadolinium chelate of diethylenetriamine pentaacetic acid bismethoxyethylamide (gadoversetamide, Mallinckrodt, St. Louis, USA), using identical long- and short-axis planes to the cine images, except for the most apical short-axis slice, which was excluded because it can be affected by partial-volume effects.

### Image analysis

All CMR studies were analyzed offline, independently, in a dedicated workstation (Viewforum; Philips Healthcare) by two similarly experienced observers (AD and GRG, both with more than six years of experience with LGE-CMR) blinded to the clinical history and patient's demographics. LV end-diastolic volume (EDV) and end-systolic chamber volume (ESV) were calculated using the Simpson method and LVEF was calculated as [EDV-ESV]/EDVx100. Basal image position was defined as the basal-most image encompassing at least 75% of the circumferential myocardium. Myocardial mass was obtained on the basis of end-diastolic endocardial and epicardial contours, and calculated as the product of myocardial volume and specific density of myocardial tissue (1.05 g/mL).

**Figure 1:**
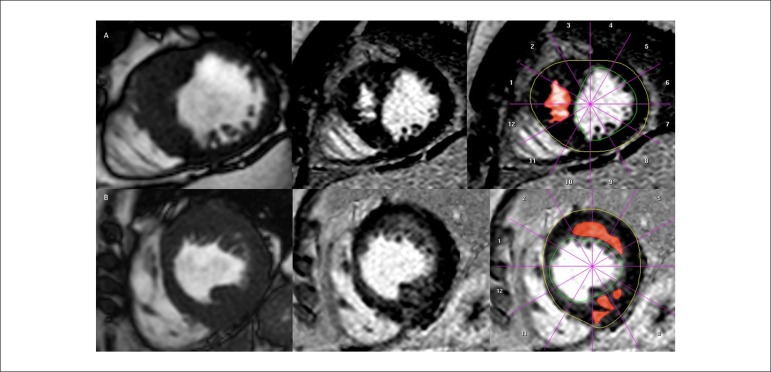
Assessment of the extent of late gadolinium enhancement (LGE) in patients with different patterns of LGE. Short-axis end diastole cine (left), gray-scale LGE images (mid panels), and segmentation defining endocardial and epicardial borders (right) to establish the myocardial volume, excluding the papillary muscles and left ventricle blood pool. Subsequently, LGE-positive regions are manually determined adjusting a gray-scale threshold to define areas of visually identified LGE (right). These areas were then summed across the short axis stack to generate a total volume of LGE. Above: 28-year old male, maximum thickness 25.5 mm (observer 1) and 25.6 mm (observer 2); percent LGE and number of segments with LGE 11% and 5 segments (observer 1) and 11% and 5 segments (observer 2). Below: 63-year old female, maximum thickness 19.4 mm (observer 1) and 19.0 mm (observer 2); percent LGE and number of segments with LGE 6% and 5 segments (observer 1) and 10% and 6 segments (observer 2).

Maximal LV WT was defined as the greatest thickness at any segment within the LV myocardium. At LGE-CMR imaging, LGE was defined as a significant increase in signal intensity compared to the remote myocardium. Such analysis is related a to a threshold ≥ 6 standard deviations above the mean signal intensity of remote myocardium, and is generally used as the reference standard.^8-11^ The extent of LGE was defined using the AHA 17-segment LV model, excluding the apex (AHA-segment 17) from the analysis. The extent of LGE was assessed both visually by the number of segments with LGE, and quantitatively by the total LV mass with LGE (% LGE). For this purpose, the LV endocardial and epicardial borders on LGE images were manually planimetered to define the myocardium, excluding papillary muscles and the intertrabecular blood pool (Figure 1). LGE-positive regions were manually determined adjusting a gray-scale threshold to define areas of visually identified LGE. These areas were then summed to generate a total volume of LGE and expressed as a proportion of total LV myocardium (% LGE). The pattern of LGE was defined for each segment as (predominantly) subendocardial, intramyocardial, subepicardial, or transmural (100% of WT). In case ≥ 2 different LGE patterns were observed within a single segment, only the predominant pattern was registered.

All procedures performed were in accordance with the ethical standards of the institutional research committee and with the 1964 Helsinki declaration and its later amendments. Informed consent was obtained from all individual participants included in the study.

### Statistical analysis

Continuous variables are presented as means ± standard deviations or median (interquartile range), as indicated. The interobserver and intraobserver (performed more than 5 months after the original analysis) agreements were assessed using intraclass correlation coefficients, (ICC; using a two-way random effect model, absolute agreement, and average measurement) with 95% confidence intervals, and Bland-Altman plots for continuous variables, and Cohen's kappa coefficient for categorical variables.^12^ Comparisons between groups regarding the patterns of LGE were performed using chi square tests. The Bland-Altman method was used to establish the limits of agreement. A two-sided p value of less than 0.05 indicated statistical significance. Statistical analyses were performed with use of SPSS software, version 22 (Chicago, Illinois, USA).

## Results

A total of 42 patients with HCM who completed LGE-CMR investigation between September 2013 and September 2014 constituted the study population. The mean age was 51.2 ± 17.7 years, and 28 (67 %) were male. Data regarding LV diastolic and systolic volumes, LV ejection fraction, and left atrium area are depicted in Table 1.

**Table 1:** Demographical characteristics, and left ventricular (LV) morphology and function (n = 42)

Age (years ± SD)	51.2 ± 17.7
Male (%)	28 (67%)
LV end diastolic volume (ml/m^2^ ± SD)	70.3 ± 13.7
LV end systolic volume (ml/m^2^ ± SD)	25.5 ± 10.1
LV mass (grams ± SD)	158.1 ± 52.2
LV ejection fraction (% ± SD)	64.5 ± 9.1
Cardiac index (L/min/m^2^ ± SD)	2.7 ± 0.6
Left atrium area (cm^2^ ± SD)	26.4 ± 7.9

Six hundred and seventy two LV segments were independently evaluated by two observers, with a mean regional WT of 9.9 ± 5.4 mm measured by observer 1 and of 10.5 ± 5.2 mm measured by observer 2 (ICC 0.95; 95% CI 0.93-0.96). Detailed analyses of regional WT are depicted in Table 2. An excellent agreement between observers was identified regarding the maximum WT (20.7 ± 4.2 mm vs. 20.9 ± 4.0 mm, ICC 0.95; 95% CI 0.91-0.98). Both observers identified the presence of LGE in more than 70% of cases and more than 20% of segments; with a concordant median 4 segments with LGE and 2.0% LGE identified by both observers (Table 3). There was good interobserver agreement regarding the presence of LGE, on both per patient (kappa 0.88, p < 0.0001) and per segment basis (kappa 0.72, p < 0.0001). Furthermore, good agreement was observed regarding the number of segments with LGE (ICC 0.97; 95% CI 0.94-0.99) and the % LGE (ICC 0.94; 95% CI 0.88-0.97).

**Table 2:** Regional wall thickness. Differences between observers

	**Observer 1**	**Observer 2**	**Difference**	**Relative dif.**	**ICC**
Wall thickness (n = 672), mean ± SD	9.9 ± 5.4	10.5 ± 5.2	0.62 ± 2.3	6.1%	0.95
Basal wall thickness (n = 42), mean ± SD	10.6 ± 2.3	11.1 ± 2.3	0.46 ± 1.0	4.3%	0.94
Mid wall thickness (n = 42), mean ± SD	11.0 ± 2.4	11.5 ± 2.6	0.54 ± 1.3	4.8%	0.92
Apical wall thickness (n = 42), mean ± SD	7.3 ± 3.1	8.3 ± 3.1	0.95 ± 1.7	12.3%	0.90
Δmax/min wall thickness (n = 42), mean ± SD	5.2 ± 2.5	5.1 ± 2.7	0.12 ± 1.1	2.3%	0.95
Maximum thickness (n = 42), mean ± SD	20.7 ± 4.2	20.9 ± 4.0	0.19 ± 1.7	0.9%	0.95

ICC: intraclass correlation coefficient.

**Table 3:** Late gadolinium enhancement extension and patterns. Differences between observers

	**Observer 1**	**Observer 2**	**Difference**	**Relative dif**	**Kappa**	**ICC**
LGE per patient (%)	30/42 (71%)	32 /42 (76%)			0.88	
LGE per segment (%)	141/672 (21%)	163/672 (24%)			0.72	
LGE (segments), mean ± SD	3.4 ± 3.2	3.8 ± 3.2	0.41 ± 1.0	11.2%		0.97
LGE (segments), median (IQR)	4.0 (0.0; 5.0)	4.0 (0.8; 6.0)				
Percent LGE (%), mean ± SD	5.1 ± 6.6	6.2 ± 7.8	1.17 ± 3.3	20.6%		0.94
Percent LGE (%), median (IQR)	2.0 (0.0; 7.3)	2.0 (0.8; 9.5)				
**LGE pattern**						0.82
Subendocardial (%)	24/141 (17%)	52/163 (32 %)				
Intramyocardial (%)	103/141 (73%)	70/163 (43 %)				
Subepicardial (%)	10/141 (7%)	32/163 (20 %)				
Transmural (%)	4/141 (3%)	9/163 (6 %)				

ICC: intraclass correlation coefficient; LGE: late gadolinium enhancement.

**Figure 2:**
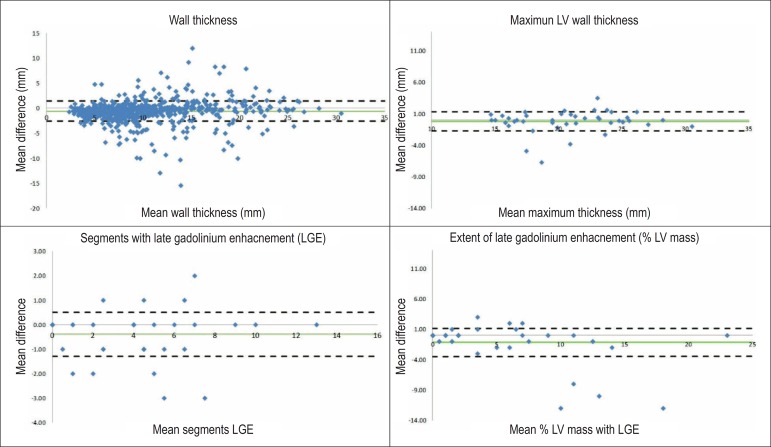
Bland–Altman plots depicting the interobserver agreement regarding mean wall thickness (panel A), maximum wall thickness (panel B), percent left ventricular mass with delayed enhancement (panel C), and number of segments with delayed enhancement (panel D). The green line represents the mean difference, and the dotted lines represent the upper (mean difference plus two standard deviations) e and lower (mean difference minus two standard deviations) limits of agreement.

The mean difference between observers and limits of agreement were as follows: 1) For WT (Figure 2a), the mean difference was -0.62 ± 1.0 mm (relative difference 6.1%), with limits of agreement of 1.36 mm; -2.60 mm; 2) for maximum WT (Figure 2b), the mean difference was -0.19 ± 0.8 mm (relative difference 0.9%), with limits of agreement of 1.32 mm; -1.70 mm; 3) for % LGE (Figure 2c), the mean difference was -1.17 ± 1.2% (relative difference 21%), with limits of agreement of 1.16%; -3.49%; and 4) for the number of segments with LGE (Figure 1d), the mean difference was -0.40 ± 0.45 segments (relative difference 11%), with limits of agreement of 0.50 segments; -1.31 segments. Conversely, there were significant differences in LGE patterns between observers, despite the fact that most patterns were judged intramyocardial by both (Table 3). Finally, there was good agreement between observations (observer 2) both regarding WT and LGE extension, whereas small differences were identified regarding the LGE patterns (Table 4).

**Table 4:** Intraobserver variability. Wall thickness and late gadolinium enhancement patterns and extension

	**Observation 1**	**Observation 2**	**Difference**	**Relative dif.**	**ICC**
Wall thickness (n = 672), mean ± SD	10.5 ± 5.2	10.7 ± 5.5	0.22 ± 2.7	2.6%	0.93
					**Kappa**
LGE per patient (%)	32/42 (76%)	32/42 (76%)			1.0
LGE per segment (%)	163/672 (24%)	168/672 (25%)			0.93
LGE (segments), mean ± SD	3.8 ± 3.2	3.9 ± 3.4	0.17 ± 0.6	2.6%	0.99
Percent LGE (%), mean ± SD	6.2 ± 7.8	6.3 ± 8.0	0.1 ± 3.0	1.3%	0.96
**LGE pattern**					0.96
Subendocardial (%)	52/163 (32%)	58/168 (35%)			
Intramyocardial (%)	70/163 (43%)	66/168 (39%)			
Subepicardial (%)	32/163 (20%)	34/168 (20%)			
Transmural (%)	9/163 (6%)	10/168 (6%)			

ICC: intraclass correlation coefficient; LGE: late gadolinium enhancement; LGE: late gadolinium enhancement.

## Discussion

CMR has been established as the reference standard to evaluate (LV) morphology and function, offering advantages regarding spatial resolution and volumetric imaging, and without limitations common to other techniques such as restricted acoustic window or radiation. Furthermore, numerous studies have validated late gadolinium enhancement CMR (LGE-CMR) to identify the presence, extent, and distribution of myocardial fibrosis in patients with HCM.^13-15^ In particular, recent reports have found that the extent of fibrosis identified by LGE-CMR in patients with HCM is independently associated to an increase in SCD and to the development of end-stage HCM.^3^

The main finding of the present study was that LGE-CMR measurements had acceptable reproducibility, with average differences close to zero, narrow limits of agreement, and a very high intraclass correlation coefficient between observers. As expected, excellent agreement was observed regarding the maximum WT. Furthermore, there was good agreement regarding the presence of LGE on both per patient and per segment basis, with the identification of LGE in more than 70% of cases and in more than 20% of the segments evaluated. These results are in line with previously reported data showing a wide range of LGE in patients with HCM, between 40 and 80%. It should be stressed, however, that the percent of the total LV mass with LGE is related to patient population, CRM system and acquisition parameters, and the employed quantification technique.^2,3,6,7^ Such variability might be attributed not only to the aforementioned genetic heterogeneity that have been suggested to include more than 1400 mutations in at least 13 genes, but possibly to a previously deemed negligible interobserver variability.^16^

Indeed, it is noteworthy that in our study, relative differences reached 11% and 21% for the number of segments with LGE and for the % LGE respectively, suggesting that reporting the number of segments with LGE might be more accurate than the percent of the LV mass with LGE. Such relative differences and the acknowledgement of the limits of agreement are of outmost importance not only due to the fact that LGE-CMR is increasingly uprising as a means to improve risk stratification in patients at risk of SCD, but also since the temporal change of such measurements might potentially become a surrogate imaging endpoint in longitudinal HCM studies.

Of note, we identified significant differences between observers regarding the main LGE pattern identified in every segment. This is not very surprising considering that almost every pattern, distribution and location of LGE has been reported in HCM.^17-19^ Furthermore, a transmural pattern has been reported in up to 50 % of HCM patients.^17^ In our study, the presence of LGE was assessed visually, since it has been shown that such analysis is highly correlated to that obtained from using a threshold of equal or more than six standard deviations above the mean signal intensity of normal myocardium, and is generally used as the reference standard.^8-10^

To the best of our knowledge, this is the first study that has specifically addressed the reproducibility of LGE-CMR patterns in patients with HCM. Mikami et al.^11^ reported the interobserver variability of a number of semi-automated LGE quantification techniques in 15 patients with HCM. Furthermore, Harrigan et al. explored the reproducibility of different semiautomated gray-scale thresholding techniques for quantifying LGE in a relatively large cohort of patients with HCM. Nonetheless, neither LGE patterns nor the number of segments were assessed in their study.^8^ The number of segments with LGE has gained clinical relevance, since it has been reported as a variable associated to an increased incidence of adverse events in different scenarios.^20-22^

In turn, we evaluated in 42 patients with HCM the reproducibility not only of LGE extension (using two approaches), but also of other parameters related to risk stratification including regional WT and LGE patterns both on a per patient and per segment basis. Our results might therefore aid investigators to perform precise power calculations for longitudinal studies.

A number of limitations should be acknowledged. We included a relatively small population of patients with HCM considering the large genetic and phenotypic heterogeneity of this disease. Furthermore, the significant differences found between observers regarding LGE patterns, aside from confirming the considerable heterogeneity in the phenotypic expression of the disease, might be partly related to the fact that only the most predominant pattern was registered per segment. Nevertheless, to the best of our knowledge, this is the largest study that specifically evaluated the reproducibility of LGE-CMR in HCM patients. We did not address the intraobserver variability since interobserver differences are usually larger and more clinically relevant for longitudinal studies.

## Conclusions

In this study, the assessment of the regional mean and maximum wall thickness using LGE-CMR in patients with HCM showed excellent reproducibility, whereas the extension of myocardial fibrosis was acceptably reproducible, and significant differences between observers were identified regarding LGE patterns. Importantly, relative differences reached 11% and 21% for the number of segments with LGE and for the percent LGE respectively, suggesting that reporting the number of segments with LGE might be more reproducible than the percent of the LV mass with LGE.
